# Epilepsy Cured by a Surgical Operation

**Published:** 1855-08

**Authors:** 


### *Epilepsy Cured by a Surgical Operation.*

To D. M. Reese, M. D.

Sir—To follow the common path of life, and to do so tamely and quietly, is to meet the common-place acquiescence of the world; and how many die utterly unknown to the page of history, simply because they feared more the criticisms of the world than they wished to benefit mankind by prosecuting a course of thought and action which would lead to it.

To step aside from the ordinary walk in which the masses tread, is often attended with censure—gross and inflexible denunciation—vituperation, and all the other windy hosts of weapons, not to mention a subjectedness to the imputation of being called an unmitigated liar. Somebody has said, and was it not Sir Isaac Newton? "I see a man must either resolve to put out nothing new, or become a slave to defend it."

This article will advance something decidedly new to the medical literature of America, but in doing so the writer will not "become a slave to defend it," for if the world will not believe it, the world can have recourse to the other alternative, viz., "just let it alone." When medical men write, (and too many of them do,) it is either for the purpose of proving something beneficial to the world, or that they themselves are fools. Now, when men advance things to the medical world—if it even be consummate nonsense—some blockhead or other will believe it; and if he does, and acts accordingly, why there will be "work for the sexton." This is rather a practical way of talking to those who delight in theoretical attenuations, but we are eminently practical, yet know something about theory too, but not any too much. In presenting a fact, it is not always necessary to use circumlocution to make it appear a fact. We are well satisfied in our own mind, and we have become so by experience—an experience extended through a series of years in experiments that prove beyond the adumbrance of a doubt, *the way to cure a man of Epilepsy is to castrate him.*

When the writer was attending the school of Pathological Anatomy, in the City of Liverpool, he heard a learned professor say, in speaking of Epilepsy, that "persons have been known to recover from Epilepsy after accidents happening to them which rendered it necessary to remove one or both of the testicles." "But," he went on to say, "to do this voluntarily, and for the sole purpose of curing this intractable malady, is too cruel, and some how or other at variance with, and repugnant to, our feelings." He also added: "Those who are aware of this as a remedy for Epilepsy, and have omitted making publicity of it, have doubtless been influenced by such reasons as have already been mentioned." The next day after hearing the above, when walking on the "green common" in company with a Scotch student, Mr. McGavoc, now of the British Navy, the subject of Epilepsy was theoretically canvassed. Mr. McGavoc said: "I knew a man in Dumfrice that had Epilepsy—he was 27 years old—had it from the age of 12, till he was 24; at this age he took mumps—was badly attended, and caught cold—metastasis to the testicles took place, and a surgeon castrated him, and cured both the mumps and the fits." He said: "It is three years since he was castrated, and has not had a fit since."

He also said: "I knew a filthy boy of about 16, who fell through a bridge when crossing it on horseback; he mashed one of the *testes*, and it had to be taken out; after it was removed he never had another fit, and it is now 6 or 7 years since the accident happened." After the writer returned home to the United States, and commenced his professional career in Tennessee, he visited the plantation of a Mr. H——, not many miles from Huntsville, Ala., who had a negro boy 14 years old, subject to attacks of Epilepsy. The writer, after examining the boy, asked his master if he was aware of the boy being fond of sexual intercourse. "Why, Dr.," said Mr. H——, "he has never been flogged more than 5 or 6 times in his life, and nearly every flogging has been for lechery. Sir, he is so bad on the little girls and *boys* that I often have threatened to "cut him." The writer then made known to Mr. H—— the evidences he had in favor of such a measure, which being acceded to, the operation of castration was at once performed, and the boy had no more fits, but has been perfectly sound ever since, and sold at the age of 20, 6 years after, for eleven hundred dollars.

Isaac T——, of Tennessee, had Epileptic fits from the age of 13 till he was forty-two; no remedy he could apply done him any good, till the writer castrated him. Anterior to this operation being performed, he was morbidly uxorious, to the extent of very great excess, and had two daughters. Nor did this operation upon Mr. T—— disqualify him for conjugal intercourse. His wife has had two sons within two years after the cure was effected. It is true, the sons did not resemble the daughters very strikingly! Mr. T—— did not fail to remark this; when he consulted the writer, by whom he was informed that certain operations upon the organs of generation produced certain other changes, only to be accounted for by invoking physiology and the psychological powers—he said it was perfectly clear to him, and that he understood it.

Three physicians to whom the writer has named his views concerning castration for the cure of Epilepsy, have adopted it, and each have been successful. Dr. White, of Tennessee, in 2 cases; Dr. Talbot, of Missouri, in 2; and Dr. Hacker, deceased, of Louisiana, in 1. Some may say that worms will produce Epilepsy, and that castration cannot cure worms. Now to such let us say, the Epilepsy of worms is not Epilepsy. Others will say that spiculæ of the skull produce Epileptic fits—neither is this Epilepsy. Nearly all kinds of fits, or such attacks of illness as neutralize the control of the voluntary system, are marshaled under the denomination of Epilepsy; but the writer holds, and he thinks he can show sufficient evidence to others, that a peculiar erethism of the ultimate nerves, of that plexus which governs the sexual system, can alone explain, as it is the only source of Epileptic fits. But as an explanation is not yet called for, it would seem unnecessary to offer it till it is. S. E. McKinley, M. D.

*Irwinton, Ga. May 22nd*, 1855.—*American Medical Gazette.*

[Are not women subject to epilepsy also, and would Dr. McKinley advise us to take out their ovaries: spay them? Masturbation and excessive venery, like other exhausting agencies, may occasionally, we have no doubt, cause the attack. When they do, which we believe to be seldom the case, the operation above proposed might prevent a return of the disease. That a man should be castrated, however, merely because he has epilepsy, is rather hard. It is well known that any serious shock to the system, as for instance a fall from a height, a severe bruise, or wound, any disturbing agency in fact, frequently puts a stop to the fit for a long period; sometimes cures the disease; and this may be the explanation of the successful result in the above cases.—EDITOR.]

